# Efficacy and safety of self-administered acupressure on symptoms, quality of life and nasal mucosal function in patients with perennial allergic rhinitis: study protocol for a randomized controlled exploratory trial

**DOI:** 10.1186/s12906-023-04132-3

**Published:** 2023-08-30

**Authors:** Kai Li, Wei Huang, Rui-Jian Li, Xiao-Cong Feng, Zong Chen, Shu-Yi Tan, Mei-Feng Xie, Jian-Peng Huang, Ru-Jia Liu, Yun-Ying Li

**Affiliations:** 1 The Second Clinical College of Guangzhou University of Chinese Medicine, Department of Otorhinolaryngology, Dade Road, Yuexiu District, Guangzhou, 510120 Guangdong Province China; 2 Second Affiliated Hospital of Shantou University Medical College, Dongxia Road, Jinping District, Shantou, Guangdong Province China

**Keywords:** Allergic rhinitis, Acupressure, Specific acupoints, Self-administered, Randomized controlled trial

## Abstract

**Introduction:**

Allergic rhinitis is a global health problem that can potentially be managed through acupressure. Our clinical observations have identified Allergic Rhinitis Acupressure Therapeutic (ARAT) as a novel acupressure treatment acting on specific acupoints, which may enhance the effectiveness of acupressure. Therefore, we propose a three-arm randomized controlled trial will be conducted to investigate the efficacy and safety of ARAT for perennial allergic rhinitis (PAR).

**Methods/design:**

In this trial, eligible 111 participants diagnosed with PAR will be randomly assigned to one of three groups: the ARAT group, the non-specific acupoints group, or the blank control group. The primary outcome will be the change in the total nasal symptom score, and the secondary outcomes will include: 1) changes in the scores of the standard version of Rhinoconjunctivitis Quality of Life Questionnaire (RQLQs); 2) acoustic rhinometry and anterior rhinomanometry; 3) changes in the scores of relief medication usage; 4) incidence of adverse events. Additionally, we will measure and compare the changes in cytokine levels (IL-5, IL-13, IFN-γ, and TSLP) in nasal secretions.

The RQLQs and primary outcomes will be assessed at the beginning, middle, and end stages of the treatment period, with monthly follow-ups conducted over a total of three months. The secondary outcomes and biomarkers in nasal secretions will be measured at the beginning and end of the treatment period. Any adverse events or need for rescue medication will be carefully noted and recorded.

**Discussion:**

This study may produce a new acupressure treatment prescription that is easy to learn, more targeted, and adaptable. This trial represents the first clinical investigation comparing ARAT treatment for PAR with the non-specific acupoints group and blank control group. Our data is expected to provide evidence demonstrating the safety and efficacy of ARAT for PAR patients, while also exploring the functional mechanism underlying ARAT treatment, moreover, the results offer valuable insights for healthcare professionals in managing PAR symptoms.

**Trial registration:**

Chinese Clinical Trial Registry, ChiCTR2300072292. Registered on June 08, 2023.

**Supplementary Information:**

The online version contains supplementary material available at 10.1186/s12906-023-04132-3.

## Background

Allergic rhinitis (AR) is a prevalent global health issue associated with a rising prevalence, imposing a substantial medical and economic burden. It is attributed to the immunoglobulin E (IgE)-mediated immune response of the nasal mucosa and involves various immune cells such as type 2 innate lymphocytes (ILC2s), T helper 2 (Th2) cells, follicular helper T cells, B cells, dendritic cells, and epithelial cells in its pathogenesis [1, 2]. AR is characterized by symptoms including sneezing, itching, running nose, and nasal obstruction [3], which can be classified as perennial or seasonal based on their correlation with specific pollen or mold spore exposures [3]. The prevalence of AR varies among different geographic regions, affecting 10%–40% of adults worldwide [4, 5]. Notably, the prevalence of AR has shown a progressive increase from 19% in 1990 to 32% in 2017 among Danish individuals aged 18-69 years [6], and in China, the standardized prevalence of adult AR has increased by 6.5% from 2005 to 2011 [7]. Epidemiological studies have revealed that approximately 16% of Americans are affected by AR [8].

While AR is not life-threatening, poorly controlled AR can lead to compounded effects and various comorbidities such as asthma, allergic conjunctivitis, chronic rhinosinusitis, and upper airway cough syndrome [3, 9]. Current therapeutic methods for AR management include patient education, allergen avoidance, pharmacotherapy, allergen-specific immunotherapy [1, 8], and surgical treatment [3, 4]. Although these approaches can effectively control most AR symptoms, they often present limitations such as incomplete relief, slow onset, less than 24-hour relief, and reduced efficacy with sustained use [10]. Additionally, poor adherence to pharmacotherapy is observed in a significant number of patients [1]. Approximately 20% of patients experience inadequate control of AR symptoms and continue to suffer from disruptive or troublesome symptoms [11–13]. Consequently, patients are increasingly drawn to complementary and alternative medicine as a potential solution [14]. utilizing these therapies to improve the management of perennial allergic rhinitis (PAR). In addition to adhering to standardized treatment guidelines for AR, we believe that the development of a complementary and alternative treatment approach that is easy-to-learn, self-implement, and effectiveness for patients is essential. Therefore, the efficacy and safety of acupressure will be evaluated in this trial.

Acupressure, also known as acupoint massage therapy, involves applying pressure to specific points on the body using fingertips. It is a well-established complementary and alternative therapy with a long history of use in use in treating various diseases in China. As a subtype and noninvasive alternative to acupuncture, which has been demonstrated to be effective and safe for PAR [8], acupressure is easy to administer and has been widely used for clinical treatment in mainland China, Taiwan, Hong Kong, South Korea, Germany, and Australia [15–19]. Two meta-analyses [16, 17] have indicated that acupressure for AR is both safe and effective. Previous studies have shown that acupressure is effective, safe, and well-tolerated in alleviating AR symptoms [18, 19]. Our research team has identified a specific combination of acupoints for AR, which is simple to apply and easy to learn. This treatment approach has been named Allergic Rhinitis Acupressure Therapeutic (ARAT) to facilitate recognition and recall.

Given the considerable therapeutic potential of acupressure as a daily treatment practice and with the aim of improving PAR management in daily life, we hypothesize that self-administered ARAT will provide a therapeutic, safe, and cost-effective option for patients with PAR. However, robust scientific evidence is needed to guide the clinical application of acupressure. Therefore, this trial seeks to investigate the efficacy and safety of ARAT in alleviating PAR symptoms, improving quality of life in adults with PAR, and exploring the feasibility of telehealth for PAR management. Two control groups, the non-specific acupoints group and the blank control group, will be included for comparison purposes.

## Methods

### Design

**Fig. 1:**
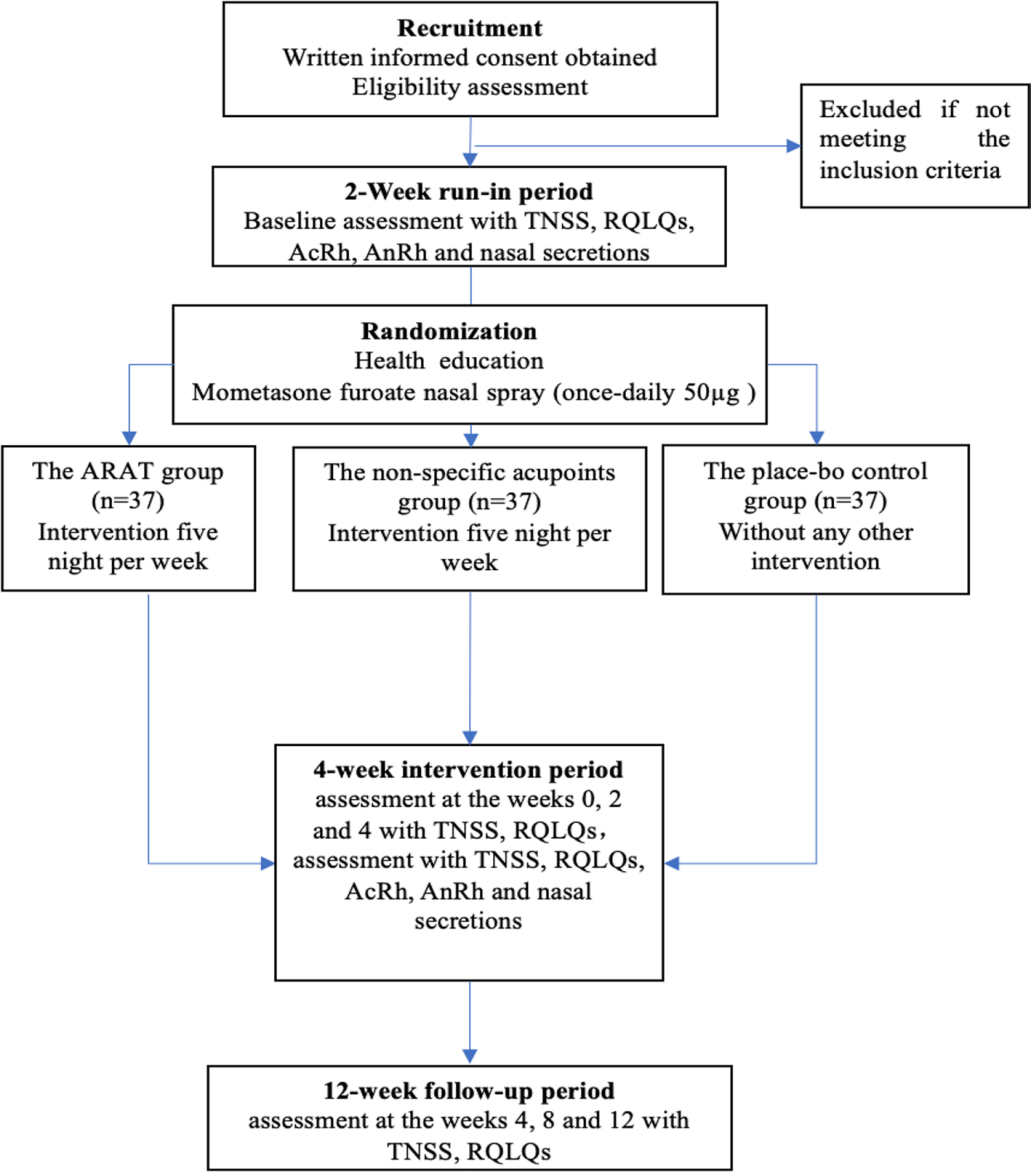
Flow chart of the clinical trial procedures. ARAT, Allergic Rhinitis Acupressure Therapeutic; TNSS, total nasal symptom score; RQLQs - Rhinoconjunctivitis Quality of Life Questionnaire score; AcRh, AcRhacoustic rhinometry; AnRh, AnRhanterior rhinomanometry

This study has been designed as a prospective, three-arm, randomized, controlled trial (RCT) and will be conducted at the Second Clinical College of Guangzhou University of Chinese Medicine (SCCGZUCM), also known as Guangdong Provincial Hospital of Chinese Medicine, in Guangzhou, China. The planned start date for this clinical trial is July 2023, and it is estimated to end by April 2024. The flowchart of the trial is provided in Fig. 1 and Table 1. The trial will consist of a 2-week run-in period, a 4-week intervention period, and a 12-week follow-up period. The study protocol has been developed in accordance with the Standard Protocol Items: Recommendations for Interventional Trials (SPIRIT) guidelines (Additional File 1) [20]. To ensure accurate interpretation and easy replication, the acupressure protocol will adhere to the Revised Standards for Reporting Interventions in Clinical Trials of Acupuncture (STRICTA), which is a formal extension of the Consolidated Standards of Reporting Trials (CONSORT) Statement [21]. Additionally, the intervention will be reported following the guide and model for Intervention Description and Replication (TIDieR) [22].

**Table 1:**
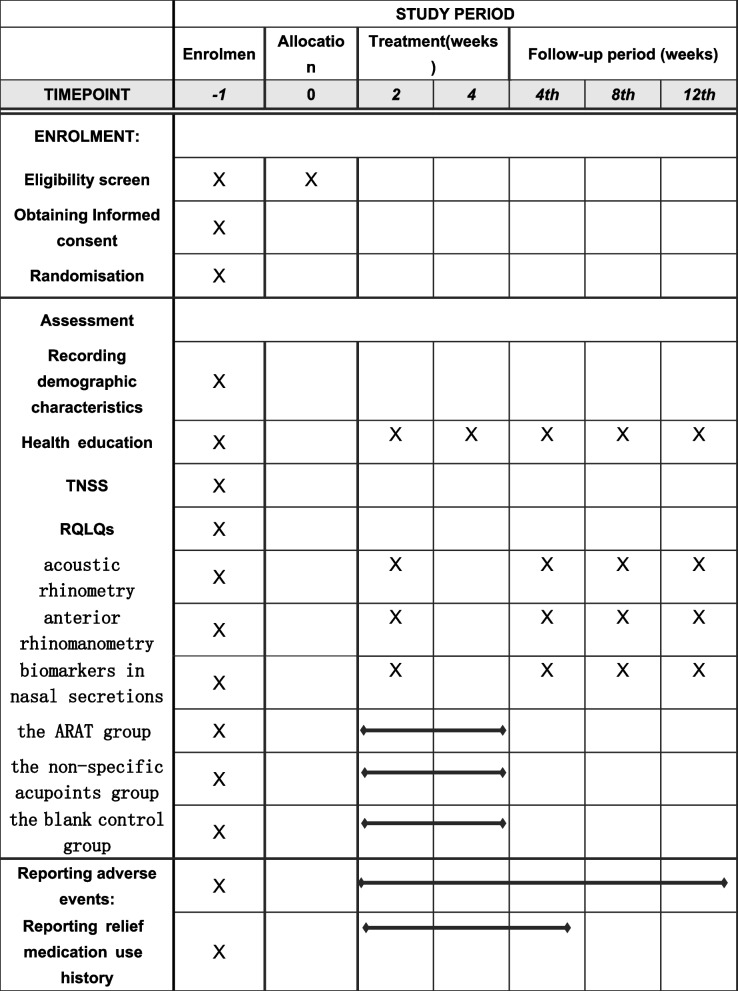
Schedule of trial enrolment, intervention, assessment and follow-up

*ARAT* Allergic Rhinitis Acupressure Therapeutic, *TNSS* Total nasal symptom score, *RQLQs* Rhinoconjunctivitis Quality of Life Questionnaire score

### Participants

Patient candidates were recruited through local media and doctor referrals. Interested candidates underwent a phone interview with our research assistants. Prior to their first visit, the study information and consent forms were sent to the participants for them to review. They were informed that nasal secretion samples would be collected. Screening evaluations were conducted and recorded during the first visit to ensure the eligibility of each participant who was willing and met the trial’s criteria. Subsequently, the research assistants obtained signed consent forms.

### Eligibility

#### Inclusion criteria

Participants must have a diagnosis of PAR according to the diagnostic criteria [3, 8, 9]; Participants must be aged between 18 and to 65; There are no gender restrictions; Participants must have the ability to communicate orally; Participants must be capable of completing the case report forms (CRF) independently or with the assistance of the investigator when necessary; Participants must volunteer to participate in the trial and provide informed consent.

#### Exclusion criteria

Participants with acute rhinitis, vasomotor rhinitis, and other non-allergic chronic rhinitis; Participants with localized skin lesions, such as burns, eczema, ulcers, or chilblains; Participants who are pre pregnant or menstruating (after menstruation, treatment can be initiated, and the date should be noted); Participants with dyssomnia or behavioral disorders that would hinder unable their cooperation with the study; Participants who are prone to being lost to follow-up; Participants using systemic or local hormones for asthma or other diseases.

#### Elimination criteria

Participants who have been enrolled in the study but cannot receive the assigned treatment at any stage due to various reasons; Participants who experience severe adverse events requiring their withdrawal from the study; Participants who do not fully participate in treatment or follow-up; Participants who do not meet the inclusion criteria but have been accidentally included; Participant who fail to comply with the treatment or provide important information for evaluation.

### Sample size

The primary outcome measure is Total Nasal Symptom Score (TNSS). As there are no existing studies on ARAT, a new type of acupressure, the sample size calculation was based on a pilot study of ARAT for PAR that utilized the same outcome measure (Table 2). Using these results and considering the study's objectives, the sample sizes for the ARAT group and the non-specific acupoints group were separately calculated for comparison with the blank control group. The calculations were performed with α=0.05 and β=0.1, and the maximum sample size for each group was determined to be 33 cases using PASS11.0 (NCSS, LLC. Kaysville, UT, USA) software. Accounting for a potential 10% dropout rate, a total sample size of 111 participants will be recruited for this trial, with 37 participants allocated to each group.

**Table 2:** Pilot study of ARAT for perennial allergic rhinitis

	**the ARAT group**	**the non-specific acupoints group**	**the blank control group**
**TNSS**			
**Pre-intervention**	9.7±2.2	9.5±2.0	10.1±1.7
**Post-intervention**	5.1±1.7^*^	7.1±1.9^*#^	7.3±1.6^*#^
**Running nose**			
**Pre-intervention**	2.5±0.5	2.4±0.7	2.6±0.5
**Post-intervention**	1.4±0.5^*^	1.7±0.5^*^	1.9±0.6^*^
**Itching**			
**Pre-intervention**	2.6±0.7	2.5±0.8	2.6±0.7
**Post-intervention**	1.5±0.8^*^	1.9±1.1^*^	1.8±0.4^*^
**Nasal obstruction**			
**Pre-intervention**	2.2±0.9	2.3±1.1	2.5±1.1
**Post-intervention**	0.8±1.0^*^	1.7±1.1^*#^	1.7±0.9^*#^
**Sneezing**			
**Pre-intervention**	2.4±0.8	2.3±1.1	2.4±0.7
**Post-intervention**	1.4±0.7^*^	1.8±0.9^*^	1.8±0.4^*^

^*^Compared with Pre-intervention *P*<0.05. ^#^Compared to the ARAT group *P*<0.05

*ARAT* Allergic Rhinitis Acupressure Therapeutic, *TNSS* Total nasal symptom score

### Randomization and blinding

#### Randomization

The randomization sequence will be generated by the Key Unit of Methodology in Clinical Research at SCCGZUCM. The random numbers will be generated using the SAS statistical analysis system PROCPLAN process statement, with the numbers generated based on given seeds. The randomization process will be managed through computer network management, and a dedicated manager who is not involved in the study will control the distribution scheme. The eligible participants will be assigned random numbers in the order of their entry into the study, and the researchers will allocate them to the treatment group or control groups accordingly.

After collecting written informed consent, the 111 participants will be randomly divided into three groups, with 37 participants in each group.

### Blinding

Considering the significant contrast in content between the treatment and control groups and the lack of an impact on the therapy's final effects, blinding procedures will not be applied in this trial. Therefore, both the investigators and the participants will be aware of the type of intervention during the clinical trials.

### Intervention

#### Health education

All eligible participants in the three groups will receive a comprehensive health education program on the introduction and management of PAR, conducted by research nurses. The program will be delivered through a video that will be accessible to participants via an app for convenient review.

#### Drugs

Participants in all three groups will be administered the same medication, which is mometasone furoate nasal spray (50μg once daily, Zhejiang Xianju Pharmaceutical Co., Ltd., Batch number: H20113481), as the routine treatment during the intervention period. Additionally, for symptom control throughout the study, participants will use cetirizine hydrochloride tablets (10mg, Chengdu LEER Pharmaceutical Co., Ltd., Batch number: H20020250).

#### Acupoints

Both in the treatment group and the non-specific acupoint group will apply self-administered acupressure to 10 specific acupoints, consisting of 7 acupoints on the head and neck and 3 acupoint on the limbs.

**Fig. 2:**
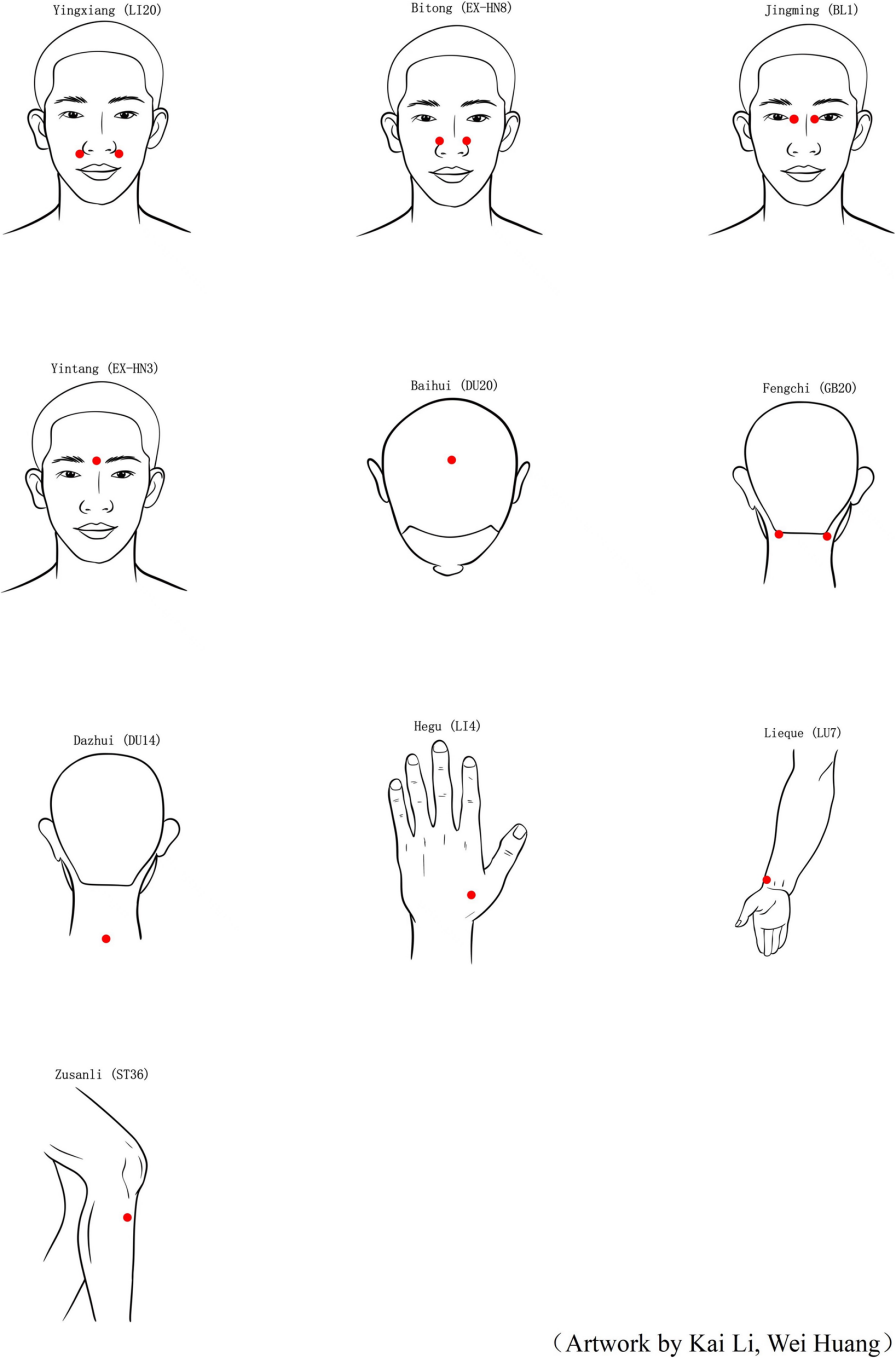
The Location of 10 acupoints of Allergic Rhinitis Acupressure Therapeutic

In the treatment group, the acupoints of ARAT, which have been used frequently used to alleviate nasal and non-nasal symptoms of PAR, will be slected for participants. These acupoints include *Yingxiang* (LI20), *Bitong* (EX-HN8), *Jingming* (BL1), *Yintang* (EX-HN3), *Baihui* (DU20), *Fengchi* (GB20), and *Dazhui* (DU14) as well as three acupoints on the limbs: *Hegu* (LI4), *Lieque* (LU7) and *Zusanli* (ST36) (Fig. 2). The selection of these acupoints is based on a review of classical and modern literature [15, 23–26].

**Fig. 3:**
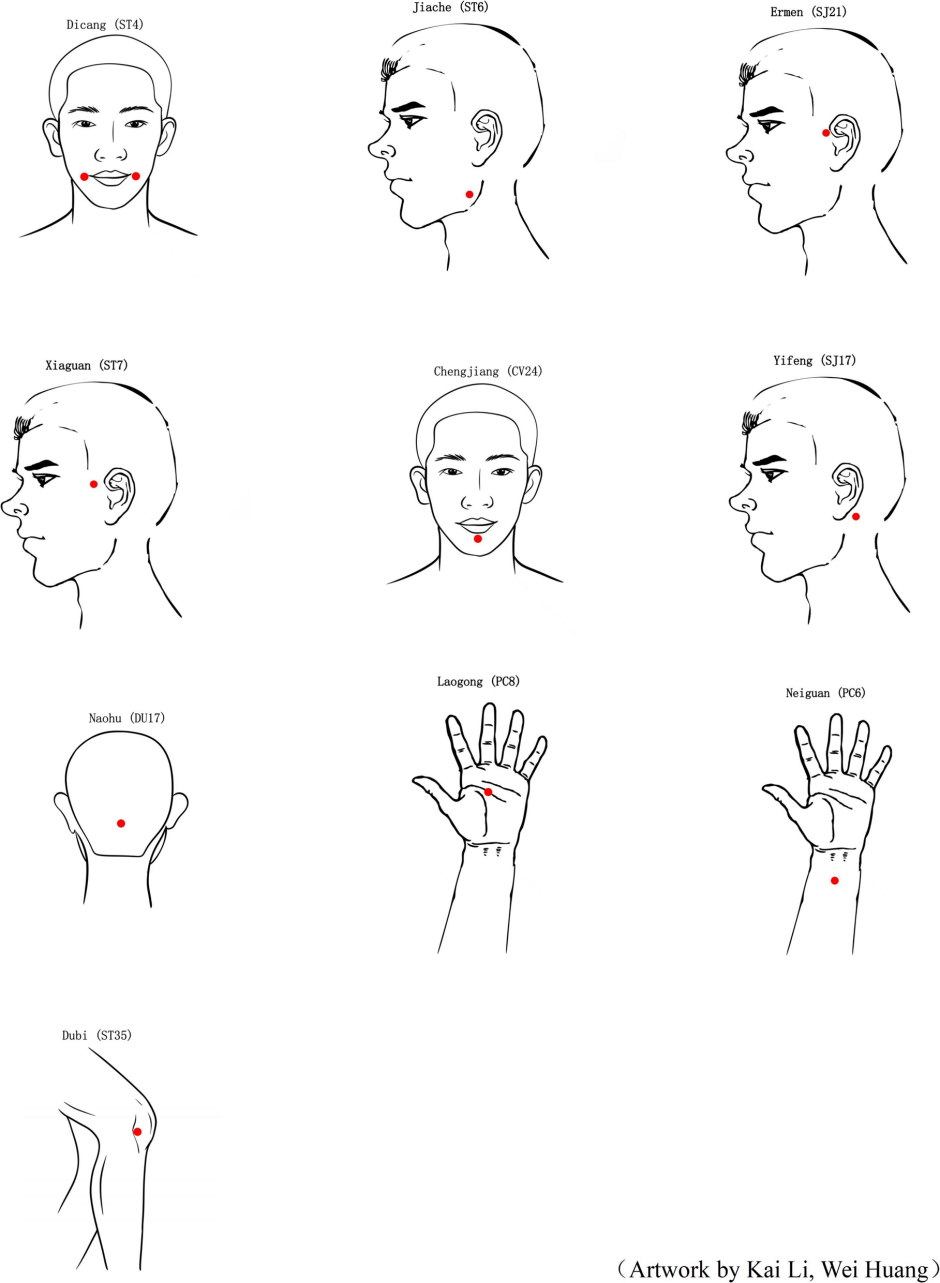
The Location of 10 Non-specific Acupoints

In the non-specific acupoint group, true acupoints that are not specifically indicated for treating AR according to the literature will be used. These acupoints include *Dicang* (ST4), *Jiache* (ST6), *Ermen* (SJ21), *Xiaguan* (ST7), *Chengjiang* (CV24), *Yifeng* (SJ17), *Naohu* (DU17), *Laogong* (PC8), *Neiguan* (PC6), and *Dubi* (ST35) (Fig. 3).

The acupoints, along with their Chinese pinyin names as per the World Health Organization (WHO) acupuncture nomenclature, can be easily located based on the provided guideline [27].

#### The treatment group (The ARAT group)

At the start of the intervention, the therapists will provide individualized, face-to-face instructions and training on ARAT techniques to each participant in the treatment group.

A video will be created that that includes lectures, demonstrations, and practice sessions conducted by nurses and therapists, demonstrating the correct acupressure techniques. This video will be uploaded to the app for participants to access.

Participants in the treatment group will perform self-administered acupressure on the designated acupoints for approximately 10 minutes every night, either after bathing or before sleeping. Participants will undergo 20 sessions treatment for 4 weeks. They will apply pressure to each acupoint 60 times, completing the entire session within the allocated time. Participants should apply sufficient pressure to experience a sensation of pressure, warmth, and slight soreness. The app will contain the video, which will be distributed to each participant. Participants will be asked to check-in at the start and end of each session.

Throughout the treatment period, participants will undergo weekly video-call check-ins to monitor their acupressure technique. Post-intervention assessments will be conducted at the second and fourth week after the intervention using the same questionnaire used in the pre-intervention assessment.

### The control groups

Participants in the non-specific acupoint group will follow the exact same procedure as the treatment group.

Participants in the blank control group will be instructed to maintain their regular daily routines, enabling the isolated effect of ARAT to be analyzed. If the results of the study are favorable, participants in the two control groups will be offered the opportunity to receive ARAT free of charge.

### Outcomes

This clinical trial will assess primary and secondary outcome measures. The outcomes will be compared between the groups at baseline, at the end of the treatment period, and at the end of the follow-up period. The recruitment, intervention, and assessment schedule for the randomized controlled trial is presented in Table 1.

#### Primary outcome measures

The primary outcome measure for this clinical trial will be TNSS. TNSS evaluates four nasal symptoms: nasal obstruction, running nose, sneezing, and nasal itch. Participants will self-assess and record the severity of their symptoms using a four-point scale (0 = no symptoms; 1 = mild symptoms; 2 = moderate symptoms; 3 = severe symptoms) [3, 28]. TNSS scores range from 0 to 12, , with lower scores indicating milder nasal symptoms. Previous studies [29, 30] have utilized the TNSS as an assessment tool.

#### Secondary outcome measures

The secondary outcome measures in this clinical trial will include the following assessments: Rhinoconjunctivitis Quality of Life Questionnaire score (RQLQs), acoustic rhinometry (AcRh), anterior rhinomanometry (AnRh), relief medication scores (RMs) and adverse events (AEs).

RQLQs is a questionnaire a questionnaire used to assess patients' quality of life, and consists of 28 questions grouped into seven domains, including activities, sleep, non-nose/eye symptoms, practical problems, nasal symptoms, eye symptoms, and emotional well-being. Each domain is ranked from no impairment to severe impairment on a scale of 0 to 6 [3, 8, 31]. The severity of symptoms during the trial will also be assessed using TNSS.

AcRh is a noninvasive and reliable method for studying the nasal cavity. It measures cross-sectional area and volume by analyzing acoustic reflections at various distances from the nasal inlet [32]. AcRh has been used to evaluate nasal dimensions in AR and assess the effectiveness of therapeutic interventions [33].

AnRh is a method designed to analyze the airflow through one nostril at a time based on trans nasal pressure. It is used to assess nasal air-space volume and patency and has become an international standard for estimating nasal resistance and flow [34].

RMs will be considered in evaluating the efficacy of the trial. The daily dose of anti-allergic medication, including antihistamines, decongestant nasal sprays, and eye drops, will be scored as 1 point. Orally administered antihistamines will be scored as 2 points, and steroid nasal sprays or eye drops will be scored as 3 points [35].

AEs will be monitored through self-reporting by the participants. Any undesirable experiences will be recorded on Case Report Forms (CRFs). Participants will have access to the research team for any concerns or issues. The occurrence time, severity, management, and causal relationship of AEs will be documented. All AEs will be followed up until resolution, and severe AEs will be promptly reported to the Ethics Committee of SCCGZUCM within 24 hours. A questionnaire will also be used to gather participants' opinions on the expectations of self-administered acupressure for AR and the credibility of the blinding method used in the study.

Additionally, the treatment mechanism of ARAT will be explored in this trial. Nasal secretions from eligible participants will be collected by research nurses at the beginning and end of the treatment. These biological specimens will be centrifuged and stored in the Biological Resource Center of SCCGZUCM for molecular analysis. Cytokines such as interleukin-5 (IL5), IL13, thymic stromal lymphopoietin (TSLP), and interferon-gamma (IFN-γ) will be detected using the enzyme-linked immunosorbent assay (ELISA) method.

Among these outcome measures, TNSS and RQLQs will be assessed at the beginning, middle, and end of treatment period, as well as during the follow-up period at weeks 4, 8, and 12. The other measures will be assessed at the beginning and end of the treatment period.

### Safety assessment

Participants will be asked to report any AEs, which are defined as any undesirable experiences [36]. They will have access to the research team members at any time. The occurrence time, severity, management, and causality of AEs will be recorded on CRFs. All AEs will be followed up until resolved, and severe AEs will be reported to the Ethics Committee of SCCGZUCM within 24 hours. Monitoring AEs in this trial serves the purpose of ensuring participant safety and dealing with adverse events in a timely manner, in addition to being a secondary outcome measure.

### Quality control and data management

To ensure adherence to the study protocol and familiarity with the administration process, all members involved in this program, including therapists, operational assistants, nurses, and statisticians, underwent systematic training before the trial commenced. The therapists responsible for applying the treatment possess Chinese medicine licenses, and both the therapists and nurses are familiar with the location of acupoints. All relevant information is recorded in CRFs, and the TNSS and RQLQs scales are carefully collected. Data entry follows the double-entry method using EpiData 3.1 software, and regular checks are performed to maintain data quality, with modifications recorded by a research assistant. Another research assistant conducts a double check of the data for any discrepancies and promptly notifies the manager if any are found. These two assistants are independent from the sponsor and have no competing interests with the authors. After repeated audits and logical checks to ensure the accuracy of the database, two sets of data will be locked and backed up for analysis. If participants withdraw from the trial at any stage, their reasons will be recorded, and the dropout rate will be analyzed statistically.

### Statistical analysis

Qualified statisticians who are blinded to the treatment group data will perform all efficacy and safety analyses following the intention-to-treat (ITT) principle using SPSS 20.0 (IBM SPSS Inc., Armonk, New York, USA). Missing values will be estimated using the last recorded data. A significance level of α= 0.05 will be considered statistically significant. The median, minimum, and maximum values will be used to express measured data. Group comparisons will be analyzed using Analysis of Variance (ANOVA) and pairwise comparisons (rank-sum test for univariate variance). Count data will be expressed as composition ratios or rates, and comparisons between groups will use the χ2 test for efficiency and rank-sum test for efficiency and rank-sum test for ranked data.

### Ethical approval

The study protocol complied with the ethical standards described in the Declaration of Helsinki. All methods of this study will be performed in accordance with the relevant guidelines and regulations. The Ethics Committee of Guangdong Provincial Hospital of Chinese Medicine has approved the study protocol for this trial (Approval number: ZF2023-040-01), and it has been registered on the Chinese Clinical Trial Registry (Trial registration number: ChiCTR2300072292. Registered on June 08, 2023). Each participant will have sufficient time to decide whether to participate in the trial or not and written informed consent must be obtained before randomization. Cetirizine Hydrochloride tablets will be provided if AR attacks interfere with participants' quality of life. Participants will be required to document relief medication use in their diaries. Any alterations or modifications to the study protocol must be communicated to the Ethics Committee of Guangdong Provincial Hospital of Chinese Medicine, Guangzhou, China, and reported to the Chinese Clinical Trials Registry. Written informed consent will be obtained from the patients and be aware of the potential benefits and risks.

## Discussion

In China, a multicenter study found that the majority of AR patients have persistent symptoms with or without seasonal aggravation [37]. Previous trials evaluating the effect of acupressure on AR did not differentiate between SAR and PAR. Considering of the characteristics of AR, only PAR patients clinically diagnosed with symptoms lasting for at least 6 months per year will be enrolled in this trial.

Intranasal corticosteroids are considered the first-line pharmacological treatment for AR due to their safety, fast action, and ability to alleviate symptoms, decrease mucus membrane permeability, and improve quality of life [1, 38]. Two long-term studies [39, 40] evaluating the safety of mometasone furoate nasal spray in children or adolescents with AR yielded positive results. Therefore, mometasone furoate nasal spray will be used as the routine treatment in this trial.

Acupuncture, a long-standing non-pharmacological treatment for AR, has been recommended as an effective alternative for symptom alleviation, improved quality of life, and reduced medication side effects [41], with supporting evidence from numerous randomized clinical trials.

Mechanisms of acupuncture for AR have also been extensively studied. Lei RL and colleagues [19] demonstrated that acupuncture exhibits anti-inflammatory and antihistaminic effects by effects by downregulating proinflammatory cytokines and neuropeptides. A recent review [42] summarized that acupuncture modulates anti-inflammatory actions in AR through various pathways, including upregulating Th1 cytokines, downregulating Th2 and proinflammatory cytokines, and affecting proinflammatory neuropeptides and neurotrophies. Clinical studies on AR patients have suggested that acupuncture treatment improves the balance between T-helper 1 (Th1) and T-helper 2 (Th2) cell-derived pro-inflammatory versus anti-inflammatory cytokines, as evidenced by differential gene expression in peripheral blood before and after acupuncture therapy [26].

Acupressure, a noninvasive and alternative form of treatment, involves applying pressure to acupoints on the body. Acupoints correspond to different organs and systems in the body [43]. Acupressure is more precise than massage, which targets broader areas to soften tissues. These advantages may align more closely with patient preferences, potentially improving adherence. Several studies have indicated that acupressure can effectively alleviate AR symptoms such as sneezing, itching, rhinorrhea, and nasal obstruction, while also enhancing quality of life [15]. In addition to its ease of use, acupressure avoids the skin irritation associated with herbal patch therapy by stimulating specific acupoints through mechanical pressure, without the use of medications.

How dose acupressure affect AR? The pathogenesis of AR in the nasal mucosa involves a complex network of inflammatory responses, encompassing various cells, mediators, cytokines, chemokines, and neuropeptides [3, 8, 9]. These phenomena are governed by cellular and molecular mechanisms that include the delicate balance between innate immune mechanisms and Th1-Th2 imbalance. Th1 T-cells primarily release IFN-γ, which plays a role in delayed hypersensitivity immune reactions [44]. TSLP, an epithelial-derived cytokine similar to IL-17, is known to induce deregulation of Th2 responses and increase the expression of chemokines that attract Th2 cells, a hallmark feature of allergic inflammatory diseases such as AR and asthma [45]. Th2 cells contribute to IgE synthesis and affect accessory cells like eosinophils, basophils, and mast cells. IL-5, an important Th2 cytokine associated with AR, can upregulate the expression of adhesion molecules, facilitating the recruitment of inflammatory cells into nasal and bronchial tissues [46]. IL-13, another key Th2 cytokine related to AR, is linked to decreased epithelial barrier integrity mediated by activated Th2 cells and may influence mucosal barrier permeability [47]. Nasal secretions, comprising cells, plasma exudation, and mucus from goblet cells and seromucous glands, will be collected bilaterally from each participant upon enrollment in the trial. The trial will observe, record, and compare the changes in the levels of IL-5, IL-13, TSLP, and IFN-γ in the nasal secretions to explore their variations.

To the best of our knowledge, this is the first clinical trial of comparing self-administered specific acupoint acupressure group and a blank control group. Additionally, it is the first trial to monitor the process online. Blinding of researchers and participants is not possible due to the nature of acupressure; however, participants will be blinded during the entire randomization process. Although non-specific acupoints have not been reported for the treatment of AR, investigation is needed to determine the therapeutic effects of acupressure on these non-specific acupoints in this trial. This trial also represents the first attempt to explore the mechanism of acupressure for PAR by analyzing and comparing cytokines in nasal secretions. It is expected to contribute valuable knowledge to fill the gaps in research. Compared to previous studies on acupressure for PAR, this trial features several innovations and advancements, including clearer diagnostic criteria for PAR classification and a more rigorous methodology. To ensure a well-designed trial, the following aspects were considered: random allocation to prevent selection bias and assessment of trial outcomes using both clinician-administered and self-reported measures.

There are certain limitations in this trial. First of all, a sham acupoint control group will not be established due to difficulties in defining sham acupoints. Secondly, individual differences in the sensitivity of acupoints and the applied pressure strength are not uniform, warranting a larger sample size to identify patterns in acupressure for AR. In this protocol, we have described a three-arm, randomized, controlled trial to examine the safety and efficacy of ARAT in treating PAR. This study will provide reliable information on the therapeutic effects of ARAT and help elucidate its potential immunologic mechanism.

In conclusion, this trial offers a standardized and practical protocol to guide current and future research. Furthermore, it contributes to the accumulation of clinical experience regarding acupressure on specific acupoints. By investigating the effectiveness and safety of acupressure, the findings of this trial are expected to provide positive evidence in support of its use for PAR patients. Moreover, the results will offer valuable insights for healthcare professionals in managing PAR symptoms.

## Trial status

Recruitment began on July 1, 2023, and is anticipated to end on April 30, 2024.

### Supplementary Information

**Additional file 1.** SPIRIT 2013 Checklist: Recommended items to address in a clinical trial protocol and related documents*.

## Data Availability

Following the completion of the present research, the datasets used and/or analyzed will be made accessible to the corresponding author upon reasonable request.

## Abbreviations

AcRh: Acoustic rhinometry
AEs: Adverse events
ANOVA: Analysis of Variance
AnRh: Anterior rhinomanometry
AR: Allergic rhinitis
CONSORT: Consolidated Standards of Reporting Trials
CRF: case report forms
ELISA: Enzyme-linked immunosorbent assay
IFN-γ: Interferon -gamma
IgE: Immunoglobulin E
IL5: Interleukin-5
ILC2s: Type 2 innate lymphocytes
ITT: Intention-to-treat
RCT: Randomized, controlled trial
RMs: Relief medication scores
RQLQs: Rhinoconjunctivitis Quality of Life Questionnaire score
SCCGZUCM: The Second Clinical College of Guangzhou University of Chinese Medicin
SPIRIT: Standard Protocol Items: Recommendations for Interventional Trials
STRICTA: Revised Standards for Reporting Interventions in Clinical Trials of Acupuncture
Th2: T helper 2
TIDieR: Intervention Description and Replication
TNSS: Total nasal symptom score
TSLP: Thymic stromal lymphopoietin
WHO: World Health Organization
